# Innate sensing of retroviral assembly by tetherin

**DOI:** 10.1186/1742-4690-10-S1-O35

**Published:** 2013-09-19

**Authors:** Stuart Neil

**Affiliations:** 1 King’s College London, UK

## 

Tetherin/BST2 is a host antiviral membrane protein that restricts the release of diverse enveloped viruses from infected cells. In the case of primate lentiviruses, virally encoded countermeasures antagonize tetherin function, promoting nascent virion release. The ability of these countermeasures to adapt to different primate species’ tetherins appears to have been important for the cross-species transmissions, in particular the zoonoses of SIVcpz that led to the appearance of the different groups of HIV-1 in humans. We are interested in the role that tetherin restriction plays in the wider antiviral immune response to lentiviruses *in vivo* and have recently found that human tetherin can transduce an NFkB-dependent proinflammatory signal upon virion retention. In this talk I will present data on the mechanism by which tetherin-mediated signaling is induced and its counteraction by HIV-1 Vpu.

